# Culture-independent genomic characterisation of *Candidatus* Chlamydia sanzinia, a novel uncultivated bacterium infecting snakes

**DOI:** 10.1186/s12864-016-3055-x

**Published:** 2016-09-05

**Authors:** Alyce Taylor-Brown, Nathan L. Bachmann, Nicole Borel, Adam Polkinghorne

**Affiliations:** 1 Centre for Animal Health Innovation, Faculty of Science, Health, Education and Engineering, University of the Sunshine Coast, Sippy Downs, QLD 4556 Australia; 2 Institute of Veterinary Pathology, University of Zurich, Winterthurerstrasse 268, 8057 Zurich, Switzerland

**Keywords:** *Chlamydia*, Culture-independent sequencing, Genomics, Reptile

## Abstract

**Background:**

Recent molecular studies have revealed considerably more diversity in the phylum *Chlamydiae* than was previously thought. Evidence is growing that many of these novel chlamydiae may be important pathogens in humans and animals. A significant barrier to characterising these novel chlamydiae is the requirement for culturing. We recently identified a range of novel uncultured chlamydiae in captive snakes in Switzerland, however, nothing is known about their biology. Using a metagenomics approach, the aim of this study was to characterise the genome of a novel chlamydial taxon from the choana of a captive snake. In doing so, we propose a new candidate species in the genus *Chlamydia* (*Candidatus* Chlamydia sanzinia) and reveal new information about the biological diversity of this important group of pathogens.

**Results:**

We identified two chlamydial genomic contigs: a 1,113,073 bp contig, and a 7,504 bp contig, representing the chromosome and plasmid of *Ca*. Chlamydia sanzinia strain 2742-308, respectively. The 998 predicted coding regions include an expanded repertoire of outer membrane proteins (Pmps and Omps), some of which exhibited frameshift mutations, as well as several chlamydial virulence factors such as the translocating actin-recruitment phosphoprotein (Tarp) and macrophage inhibition potentiator (Mip). A suite of putative inclusion membrane proteins were also predicted. Notably, no evidence of a traditional chlamydial plasticity zone was identified. Phylogenetically, *Ca*. Chlamydia sanzinia forms a clade with *C. pneumoniae* and *C. pecorum*, distinct from former “*Chlamydophila*” species.

**Conclusions:**

Genomic characterisation of a novel uncultured chlamydiae from the first reptilian host has expanded our understanding of the diversity and biology of a genus that was thought to be the most well-characterised in this unique phylum. It is anticipated that this method will be suitable for characterisation of other novel chlamydiae.

**Electronic supplementary material:**

The online version of this article (doi:10.1186/s12864-016-3055-x) contains supplementary material, which is available to authorized users.

## Background

The *Chlamydiae* are a phylum of intracellular bacteria that are characterised by their unique biphasic lifecycle [1–3]. While they are ubiquitous in the environment [4], a significant number are also associated with disease in a wide range of hosts [2]. The traditional family in this phylum, *Chlamydiaceae*, consists of a single genus, *Chlamydia*, and includes important human and animal pathogens such as *Chlamydia trachomatis* and *Chlamydia psittaci*. Even though this is the best-understood family in the phylum, two new species, and one *Candidatus* species, in the genus *Chlamydia*, were recently described from birds [5, 6], highlighting how little we still know about the full diversity of these obligate intracellular pathogens. Aside from human and mammalian hosts [7], chlamydiosis has been reported in both free-ranging and captive reptilian hosts including several snake species, turtles, tortoises and crocodiles, among others [8–11]. The most common species found in these hosts to date is *Chlamydia pneumoniae*, however, a recent study also revealed 16S rRNA sequences corresponding to potentially novel *Chlamydia* species [11].

Barriers for characterising the biology of these unique intracellular parasites lie in the fact they require a host cell to undergo replication. For many novel species, in vitro culture systems are not available. With deep sequencing becoming faster, more affordable and higher throughput [12], groups within the chlamydia field have recently developed several methods of deep sequencing from clinical samples to gain insight into the biology of these species [13–17]. These alternative methods bypass the labour-intensive and costly culture step that has hampered genomic characterisation of novel pathogens_._

In the current study, we have utilised a culture-independent method to sequence the genome of a previously uncharacterised and uncultivable new member of the genus *Chlamydia*. The subsequent comparative genomics enabled by this approach allows for fast and effective identification of a number of hallmarks of chlamydial biology in these novel species, including virulence factors and membrane proteins.

## Results and discussion

### Metagenome reconstruction and chlamydial genome assembly

5,561,445 paired reads were obtained from the treated DNA sample following shotgun sequencing on an Illumina MiSeq. As no reference genome was available for this putative novel species, reads were trimmed prior to *de novo* assembly into 261,306 contigs. BLASTn analysis revealed two contigs that were suspected to be of chlamydial origin: a 1,113,073 bp chromosomal contig with 81 % nucleotide identity with *C. pneumoniae* LPCoLN (CP001713.1), and a 7,504 bp plasmid contig with 77 % nucleotide identity with the plasmid of *C. pneumoniae* LPCoLN (CP001714.1) (Additional file 1: Table S1). 329,886 reads mapped to the chromosomal contig, accounting for ~6 % of the reads (Additional file 1: Table S2). The mean read coverage across the genome was ~44×, with at least 10x read coverage at every base with the exception of some of the predicted polymorphic membrane proteins. Interestingly, the mean coverage of the plasmid was estimated to be ~1888x, with the plasmid reads accounting for ~37 % of the total reads.

Despite treatment of the DNA to enrich for microbial DNA, at least 227,252 contigs are believed to be host-derived, based on BLASTn analysis against two available snake genomes (*Python bivittatus*; AEQU00000000.2 and *Pantherophis guttatus;* JTLQ00000000.1), although the majority of these contigs are very short (Additional file 1: Table S2, Table S3). While the method used to enrich for microbial DNA relies on depletion of methylated DNA, there is evidence to suggest that vertebrate mitochondrial DNA may not be methylated at many regions [18]. This would explain the high coverage (~1904×) observed for the mitochondrial genome obtained in this metagenome, accounting for ~5 % of the reads. (Additional file 1: Table S2).

The metagenome was also screened for host-associated microflora to assess the proportion of reads devoted to other bacterial species. Full or partial 16S rRNA sequences were detected for five non-chlamydial bacteria in the sample, with significantly lower coverage than the chlamydial genome. (Additional file 1: Table S4).

Automated annotation of the chlamydial chromosome and plasmid by RAST followed by manual annotation in Artemis resulted in prediction of 998 coding regions, demonstrating a similarly reduced gene content to other members of the *Chlamydiaceae* (Table 1). Roughly one third of the genome consists of hypothetical proteins (314). The two chlamydial genomic contigs of uncultured *Chlamydia sp*. 2742-308, had G + C contents of 38.5 % and 32.3 %, respectively (Table 1) [5, 6]. The chromosome was shown to be able to be circularised *in silico* by read mapping across the contig break (Additional file 2: Figure S1).

**Table 1:** Comparative analysis of chlamydial genome features. One strain representative for each species was analysed

*Species*, *strain* (*accession number*)	*Ca.* C. sanzinia 2742-308 (CP014639)	*C. pneumoniae* LPCoLN (CP006571.1)	*C. pecorum* MC/MarsBar (NZ_CM002310.1)	*C. psittaci* 6BC (NC_017287.1)	*C. felis* Fe/C-56 (AP006861.1)	*C. caviae* GPIC (AE015925.1)	*C. abortus* S26/3 (NC_004552.2)	*C. avium* 10DC88 (CP006571.1)	*C. gallinacea* 08-1274/3 (CP015840.1)	*Ca. C. ibidis* 10-1398/6 (APJW00000000.1)	*C. muridarum Nigg* (NC_002620.2)	*C. trachomatis* A/HAR-13 (NC_007429.1)	*C. suis* MD56 (CM002267.1)
Chromosome length (Mbp)	1.11	1.24	1.11	1.17	1.17	1.17	1.14	1.04	1.05	1.15	1.07	1.04	1.07
GC content (%)	38.5	40.5	41.1	39.1	39.4	39.2	39.9	36.9	37.9	38.3	40.3	41.3	42.0
No. CDSs	998	1097	945	1003	1005	998	964	940	898	949	904	911	915
Hypothetical proteins	314	426	297	337	324	376	219	242	207	235	353	294	218
Plasmid length (Kbp) (No. ORFS)	7.5 (8)	7.5^a^ (8)	7.5^a^ (8)	7.5 (8)	7.5 (8)	7.9 (7)	Np	7.1 (7)	7.0 (7)	Np	7.5 (8)	7.5^b^ (8)	5.9 (6)

*Np* No plasmid

^a^Plasmid not present in all strains

^b^Plasmid length of *C. trachomatis* L2b/CS784/08

### Description of “*Candidatus* Chlamydia sanzinia”, sp. nov

Based on the novel nature of the assembled chlamydial genome sequenced in this sample, we propose for it a novel species with Candidatus status in the genus *Chlamydia,* in the absence of maintenance in culture: *Ca.* Chlamydia sanzinia (*Sanzinia,* pertaining to the host genus name). *Ca.* C. sanzinia shares < 97 % 16S rRNA nucleotide identity with other *Chlamydia spp*. Additionally, it shares < 96 %, <98 %, <96 %, <95 % and <95 % nucleotide sequence identity with other *Chlamydia spp*. for *rpoN, ftsK*, *pepF*, *adk* and *hemL*, respectively (Additional file 1: Table S5); classifying it as a new species using the scheme recommended by Pillonel et al. [19]. This novel species was detected in the choana of a captive asymptomatic Madagascar tree boa (*Sanzinia madagascariensis volontany*) in a private collection in Switzerland.

### *Ca*. Chlamydia sanzinia forms a distinct clade with *C. pneumoniae* and *C. pecorum*

**Fig. 1:**
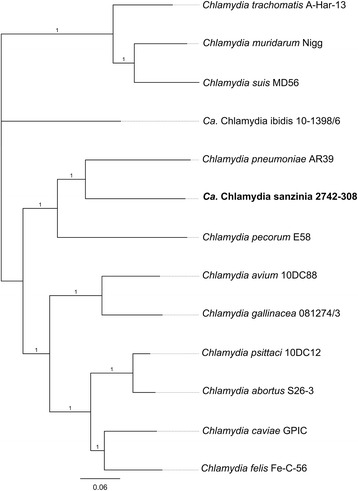
*Chlamydia* species core genome phylogenetic tree. The core genome was extracted using the LS-BSR package and phylogenetic tree constructed using FastTree. Numbers on the branches indicate support values

Using the novel sequence data for this unique chlamydial taxon, we extracted the core genome for comparative phylogenetic analyses to show that *Ca.* Chlamydia sanzinia is most closely related to *C. pneumoniae* (LPCoLN strain), with an average nucleotide identity of 76.9 %, in agreement with the 16S rRNA sequence identity previously described for this putative novel species [11]. In our phylogenetic tree construction, this taxon shares the same minor clade with *C. pneumoniae* and *C. pecorum*, within a major clade comprised of former “*Chlamydophila*” *spp.* and the novel avian *Chlamydia spp* and distinct from *C. muridarum*, *C. suis* and *C. trachomatis* (Fig. 1). Branch lengths indicate that *Ca.* C. sanzinia and *C. pneumoniae* have evolved at a similar rate after divergence from a common ancestor.

### The genome of *Ca.* Chlamydia sanzinia harbours a 7.5 Kbp cryptic plasmid

**Fig. 2:**
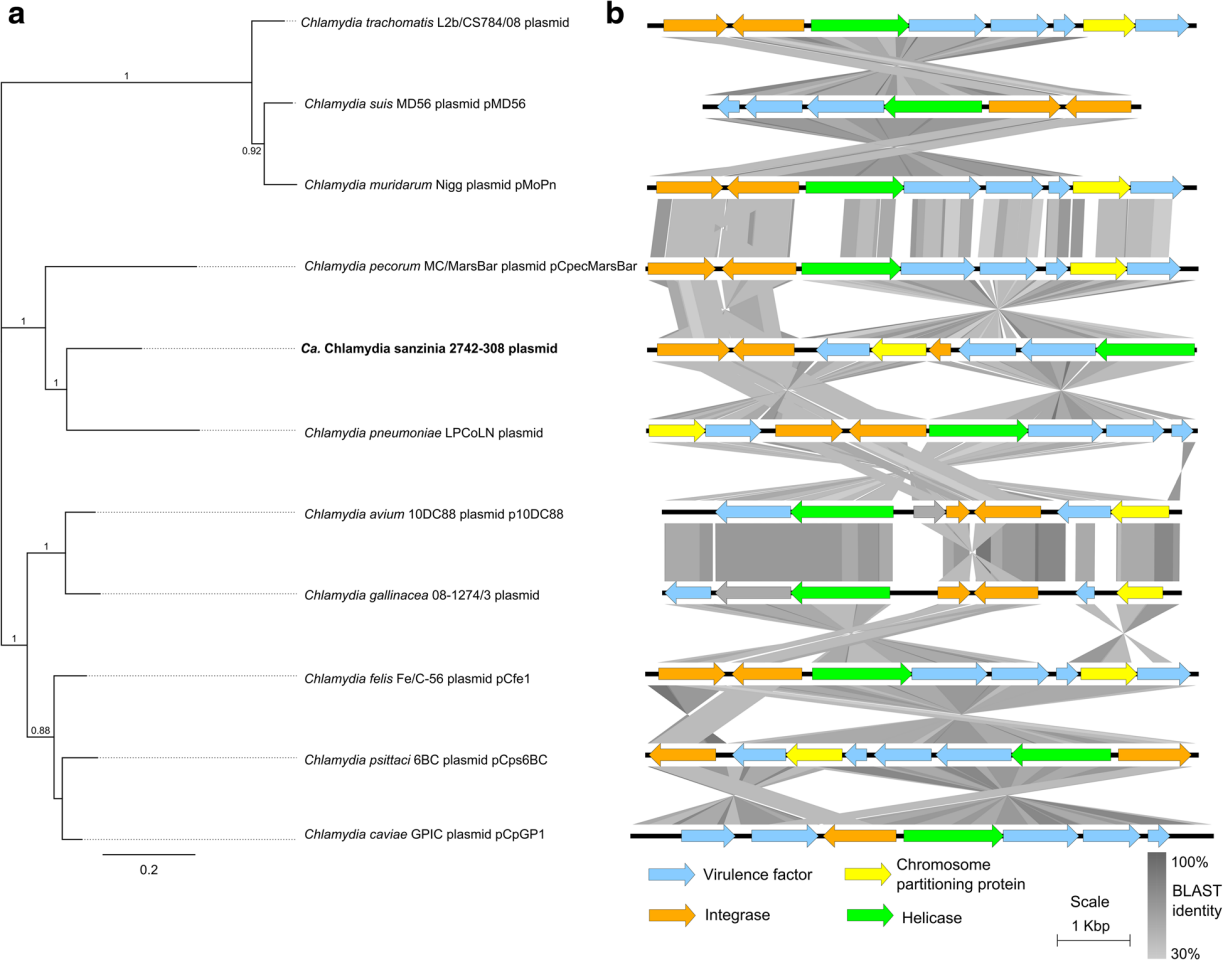
Chlamydial plasmid phylogeny and arrangement. (**a**) Chlamydial plasmid proteins were extracted from each sequence, concatenated and aligned prior to phylogenetic tree construction using the FastTree algorithm in Geneious; (**b**) *Chlamydia* plasmid nucleotide sequences were compared via tBLASTx analysis and their arrangement plotted in EasyFig *Block arrows* represent proteins, coded by colour and *grey shading* represents sequence homology

Chlamydial plasmids are nonconjugative, nonintegrative and found in most chlamydial species [19]. While we are still understanding the function of this plasmid, studies have suggested roles in regulation of the developmental cycle and in influencing tissue tropism and disease outcome [20-22]. *De novo* assembly of the trimmed reads produced an extra-chromosomal contig resembling the chlamydial plasmid, the gene content and arrangement of which are typical of other chlamydial plasmids (Fig. 2b). The *Ca.* C. sanzinia plasmid proteins share 42.7 to 87.2 % amino acid similarity with *Chlamydia spp.* plasmid proteins (Additional file 3: Tables S6–S13). The phylogenetic tree constructed from a concatenated alignment of the conserved proteins, reflects these relationships (Fig. 2a), which mirror that of the chromosome. The nucleotide sequence identity across the plasmid was much lower, ranging from 31 to 69 %, although much of this variation can be attributed to genes absent from some of the plasmids (Table 1, Fig. 2b).

It is also interesting to note that coverage of the plasmid contig was almost 43 times that of the chromosome (~1,888× vs ~44× and accounting for ~37 % of the reads), suggesting a plasmid copy number of up to 43 per chromosome. This seems unlikely given previous descriptions of plasmid copy numbers in the range of two to ten [23–25]. An alternative explanation for the high coverage is that the MDA process preferentially amplifies the plasmid. Other studies have also shown that plasmid copy numbers change throughout the developmental cycle [23–25].

### The genome of *Ca.* Chlamydia sanzinia does not appear to contain a plasticity zone

The chlamydial plasticity zone (PZ) is a region of extensive variation between chlamydial genomes [2, 7], which, while highly variable, generally harbours (i) acetyl coA carboxylase chains (*acc*BC), (ii) cytotoxin genes/adherence factor, (iii) phospholipase D (PLD), (iv) membrane attack complex/perforin (MACPF), (v) tryptophan biosynthesis operon (*trp*ABFCDR, *kyn*U, *prs*A) and (vi) purine biosynthesis genes (*gua*AB*-add*) [2, 7]. Iterations of the chlamydial PZ have been described in the genomes of all species of *Chlamydia* to date ranging from ~12 kbp to ~86 kbp, with 11 to 48 genes [2, 7].

**Fig. 3:**
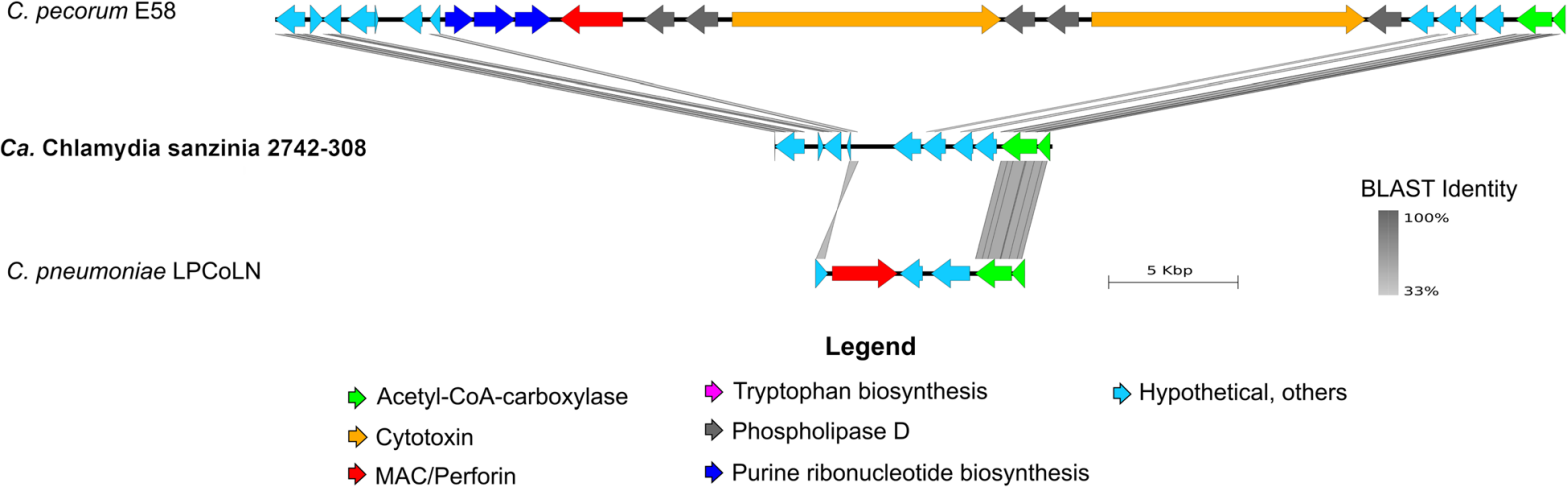
Lack of the plasticity zone in *Ca*. Chlamydia sanzinia. The region of the genome encoding the plasticity zone in *C. pecorum* and *C. pneumoniae* were compared to that of *Ca*. C. sanzinia via tBLASTx analysis and their arrangement plotted in EasyFig. *Block arrows* represent proteins, coded by colour and *grey shading* represents sequence homology

Despite rigorous homology and conserved domain searches through all 261,306 contigs, the only features of a chlamydial PZ in *Ca.* C. sanzinia are *acc*B & *acc*C (Cs308_0799 & Cs308_0800) (Fig. 3). While this region is very small, it is similar to that of its closest relative, *C. pneumoniae* LPCoLN and to that of *C. avium* 10DC88, both of which only possess *acc*BC and MACPF. Two hypothetical proteins have weak sequence similarity to *C. psittaci* adherence factor (*tox*) (Cs308_0802 and Cs308_0803). The finding of neither a MACPF or plasticity zone-PLD is consistent given the evidence that MACPF may assist PLDs in lipid acquisition and processing [2], in which case the absence of a MACPF may depend on the absence of a PLD. The lack of a complete *trp* operon, a feature shared with its closest relative, *C. pneumoniae*, as well as other *Chlamydia spp.* except *C. pecorum*, *C. caviae* and *C. felis,* suggests a different pathway for synthesising tryptophan. Interestingly, an aromatic amino acid synthase, which has been described as an alternative pathway for *trp* synthesis in *C. pneumoniae* [26], also appears to be absent. The lack or truncation of the *guaAB-add* operon for purine biosynthesis is common to *C. trachomatis*, *C. abortus*, and some strains of *C. pneumoniae* and *C. psittaci*. The genome of *Ca*. C. sanzinia does however appear to possess an AMP nucleosidase (Cs308_0522) which plays a role in purine nucleoside salvage.

We considered the possibility that the plasticity zone was either not sequenced or not assembled, given that multiple displacement amplification may introduce an amplification bias [27], as seen for the plasmid. As shown in Additional file 2: Figure S1, however, overlapping read mapping to the joined ends of the chromosomal contig demonstrates that we have obtained the complete genome for this bacterium.

The absence of an obvious chlamydial plasticity zone characteristic to other members in the genus, as such, sets this putative novel species apart from its known closest phylogenetic relatives. Strain diversity studies in other chlamydial species suggest that further investigation could elucidate remnants of a PZ if other strains of this novel taxon were to be characterised.

### An expanded repertoire of polymorphic membrane proteins are located on “pmp islands” for *Ca. *Chlamydia sanzinia

Chlamydial membrane proteins are thought to play an important role in host-parasite interaction throughout the chlamydial developmental cycle. Given their antigenic properties, many have also been the target of extensive vaccine studies [28]. Polymorphic membrane proteins (Pmps) are one such family of membrane protein, unique to chlamydiae, that are highly variable but are united by their GGA (I, L, V) and FxxN tetrapeptide motifs [28, 29]. They also function as autotransporters in the Type V secretion system [29]. Investigation of the genome of *Ca.* C. sanzinia revealed not only the presence of homologues of previously described *pmp* genes, exhibiting the aforementioned motifs, but also the presence of an expanded group of membrane proteins annotated as *omp5,* an outer membrane protein (Cs308_0070, 0072, 0079, 0084, 0085, Cs308_674-677). These protein encoding genes share significant sequence homology with either *pmp* or *omp* proteins from *C. pneumoniae*, *C. pecorum*, *C. psittaci* and *C. abortus* (43–62 % amino acid similarity). This family of membrane protein genes, together with the *pmp*s, are arranged in four clusters, not dissimilar to the *pmp* distribution in other chlamydial genomes (Additional file 4: Figure S2).

Although twelve *omp5* genes were annotated by automated methods, further analysis determined three of these (Cs308_0074, Cs308_0076 and Cs38_0077) to be fragments of two *pmpG* pseudogenes (Cs308_0073 and Cs308_0075), with truncations attributed to frameshift mutations (Additional file 4: Figure S3). The fragments together contain the repeat motifs, middle domain protein and autotransporter domains characteristic to pmps. Frameshifts are common to *pmp* encoding genes, and it has been suggested that these mutations promote antigenic diversity [26].

*Pmp* genes account for around 4 to 5 % of the coding regions in closely related chlamydial species (up to 22 % for some strains of *C. pneumoniae* [26]). The predicted Pmp-encoding genes in this genome account for 3.1 %; with the inclusion of the additional *omp5* genes these membrane proteins together account for approximately 5 % of the coding regions of this genome, suggesting the expanded *pmp* and *omp* repertoire confers characteristics advantageous to survival inside the host cells infected by this novel chlamydial agent.

### *Ca.* Chlamydia sanzinia displays genomic hallmarks of a pathogen

The *Chlamydiae* possess a number of mechanisms to exploit the host cell machinery in order to survive and replicate inside a host cell. One of the ways in which chlamydiae achieve this is by the secretion of “effectors”, virulence factors which function to influence host signalling, cleave host proteins and suppress host defences. Homologues of several virulence factors secreted by the Type Three Secretion System (T3SS) are present in the genome of *Ca.* C. sanzinia: namely, a homolog of translocated actin-recruiting phosphoprotein (Tarp) (Cs308_0200), macrophage inhibition potentiator (*mip*) (Cs308_0291), tail-specific protease (*tsp*) (Cs308_0179) and serine/threonine protein kinase (*Pkn*D) (Cs308_0709) [30], while genes encoding the chlamydial outer protein, CopB, and a chlamydial protein associating with death domains (CADD), appear to be absent [30]. Another chlamydial virulence factor, chlamydial protease-like activity factor (CPAF) (Cs308_0639), secreted by the *sec*-dependent pathway [31], was also identified in this novel genome.

The inclusion membrane proteins (Incs) are another group of membrane proteins unique to the *Chlamydiae*, exposed to the host cytosol and hypothesised to be involved in inclusion membrane biogenesis [32, 33]. They share little sequence similarity but instead possess a 40-60 amino acid bi-lobed hydrophobic structure [32]. In addition to two copies of *Inc*A and single copy each of *Inc*B and *Inc*C (Cs308_0059, Cs308_0863, Cs_0864, Cs_0885), an additional 41 putative Inc proteins were predicted *in silico* in the *Ca*. C. sanzinia genome (Additional file 5: Table S14). This is within the range described for other *Chlamydia spp*, which are predicted to encode as few as 36 Inc proteins (*C. trachomatis*) or up to as many as 107 (*C. pneumoniae*) [32], providing further evidence that these proteins are integral to inclusion development and host interaction across the genus [2].

## Conclusion

For many novel species within and outside of the *Chlamydiae*, culture-independent sequencing from clinical samples provides a unique opportunity to understand the biology of the species for which there is a) no established culture system or b) no reference genome. While our group and others have recently used several genome sequencing methods to broaden our knowledge of previously described species in the genus *Chlamydia* [13–17], the current study suggests that a shotgun deep sequencing approach is better suited to novel species. For instance, using “bait” probes designed from a reference genome risks overlooking previously undetected or undescribed features, such as a plasmid [25]. Likewise, using only homology or binning approaches for metagenomes from clinical samples such as this may also omit accessory proteins or extra-chromosomal sequences that either share no homology to known proteins or are lacking in conserved phylogenetic markers. The use of MDA for whole genome amplification prior to genome sequencing does come with its own limitations however, such as preferential amplification of certain genomic regions or particular bacteria in a microbial community [27, 34]. The amplification skew is unpredictable and appears to be heavily dependent on both the complexity of the sample and the MDA protocol applied [27]. These issues appear to be able to be partially overcome by combining MDA with other depletion or enrichment methods [15]. Nonetheless, these tools are particularly useful for uncultivable intracellular organisms such as members of the phylum *Chlamydiae*, and can likely be incredibly valuable for further characterisation of a range of novel chlamydiae reported within and outside the genus *Chlamydia*. This method also circumvents sequencing of genetic changes that are often acquired during passaging.

We have used this method to sequence and characterise a novel uncultured bacterium in the genus *Chlamydia* from the first reptilian host, expanding our understanding of the diversity and biology of a genus that was thought to be the most well-characterised in this biologically unique phylum.

## Methods

### DNA extraction and microbial DNA enrichment

Genomic DNA was extracted from a swab from the choana of a Madagascar tree boa (*Sanzinia madagascariensis volontany*) as per [11]. Total genomic DNA was then subject to host methylated-DNA depletion using the NEBNext Microbiome DNA Enrichment kit (New England BioLabs, USA), according to manufacturer’s instructions. The selectively enriched DNA was purified by ethanol precipitation before being subject to multiple displacement amplification using the Qiagen Repli-G mini kit (Qiagen, Germany), according to manufacturer’s instructions.

### Genome sequencing and assembly

Sequencing was carried out on an Illumina MiSeq at the Australian Genome Research Facility, Walter & Eliza Hall Institute, Parkville, Australia using 150 bp paired end reads. Read quality was assessed with FastQC v0.11.2 and trimmed using Trimmomatic v.035 [35]. Trimmed reads were *de novo* assembled using SPAdes v3.1.1 [36], with kmer values of 51, 71, 91, 101 and 127 in both multi-cell and single-cell mode. QUAST [37] was used to assess the quality of the assemblies (Additional file 6: Table S15).

### Genome annotation and analysis

Resulting contigs were subject to a BLASTx search against an in-house chlamydial protein database. Contigs longer than 1000 bp with hits with e-values ≤ 0.005 and identity values ≥ 60 % were subsequently also manually compared to the NCBI database. Two chlamydial contigs were uploaded to RAST [38] for automated annotation. Additional coding regions were identified and annotated using Artemis [39]. The chromosomal contig was split to resemble the genome architecture of *C. trachomatis*, and reads were mapped back to the assembly to assess genome coverage. A 60 bp region with <10x coverage was removed from the 5’ end of the contig, resulting in reads overlapping the the 5’ and 3’ ends of the contig. A similar method was also applied to the plasmid contig; a 124bp region was removed from the 5’ end. The nucleotide sequence of the genome of uncultured *Ca*. Chlamydia sanzinia strain 2742-308 was deposited in Genbank under accession numbers CP014639 (chromosome) and CP014640 (plasmid).

To identify the origins of non-chlamydial contigs, Metaxa [40] was employed to detect ribosomal RNA sequences of prokaryotic and eukaryotic origins. These results informed downstream read-mapping to assess the proportion of reads belonging to different origins using Geneious [41], BWA aligner [42], and BEDTools [43]. The visualisation of the BLAST comparisons in Figs. 2 and 3 were generated using Easyfig [44].

### Phylogenetic analysis

To assess the genome-wide phylogenetic relationships among *Chlamydia spp*., the core genome was extracted using the LS-BSR package [45]. A phylogenetic tree was constructed from the computed alignment using FastTree [46] and visualised in Geneious [41]. For the plasmid phylogeny, each gene was extracted and translated *in silico*, prior to concatenation. The phylogenetic tree was then constructed from the resulting alignment.

### Inclusion membrane protein prediction

The amino acid sequences for all 314 hypothetical proteins were subject to transmembrane helix prediction using TMHMM [47]. Hydropathy plots were visualised to identify characteristic bi-lobed hydrophobic domains.

## Additional files

Additional file 1: Table S2. Chlamydial contig BLAST analysis. **Table S3**. Metagenome coverage statistics. **Table S4.** Putative host contig analysis. **Table S5.** rRNA-containing contigs detected using Metaxa. **Table S5**. Phylogenetic marker gene BLAST analysis. (PDF 152 kb) Additional file 2: Figure S1. Contig break mapping. The chromosomal contig was split to resemble the genome architecture of *C. trachomatis*, and reads were mapped back to the assembly to assess genome coverage in Geneious. A 60 bp region with <10× coverage was removed from the 5’ end of the contig, resulting in reads overlapping the the 5’ and 3’ ends of the contig. (JPG 257 kb) Additional file 3: Tables S6–13. Plasmid protein homology. (XLSX 22 kb) Additional file 4: Figure S2. Locations of “pmp islands” in the uncultured *Ca*. Chlamydia sanzinia genome and selected chlamydial genomes. Figure was constructed in Geneious. **Figure S3.** Schematic representation of *pmp* pseudogenes attributed to frameshift mutations. Open reading frames are coloured blue in frame and genes/pseudogenes are coloured white on the forward strand. Vertical black lines represent stop codons. Diagonal blue lines represent fragmentation due to premature stop codons. Figure constructed using Artemis. (PDF 140 kb) Additional file 5: Table S14. Inclusion membrane prediction. (PDF 99 kb) Additional file 6: Table S15. QUAST report. (PDF 127 kb)
