# Supplementary material for: Culture-independent genomic characterisation of Candidatus Chlamydia sanzinia, a novel uncultivated bacterium infecting snakes
Source: BMC Genomics. 2016 Sep 5;17(1):710. doi: 10.1186/s12864-016-3055-x (PMC5011893; doi:10.1186/s12864-016-3055-x)
Supplement: Additional file 1: Table S2. — Chlamydial contig BLAST analysis. Table S3. Metagenome coverage statistics. Table S4. Putative host contig analysis. Table S5. rRNA-containing contigs detected using Metaxa. Table S5. Phylogenetic marker gene BLAST analysis. (PDF 152 kb) [file 12864_2016_3055_MOESM1_ESM.pdf]

Supplementary Table S1: Phylogenetic marker gene BLAST analysis

Gene	Top BLASTn hit (accession no.)	%ID	Top BLASTp hit (accession no.)	%ID
rpoN	*Chlamydia pneumoniae* genome assembly YK41, chromosome : 1 (LN849047)	67%	RNA polymerase sigma-54 factor (*Chlamydia pneumoniae*) [NP_224966.1] (WP_010883408)	61%
ftsK	*Chlamydophila pneumoniae* LPCoLN, complete genome (CP001713)	77%	DNA translocase FtsK (*Chlamydia pneumoniae*) [NP_225075.1](WP_010883515)	75%
pepF	*Chlamydia pneumoniae* genome assembly UZG1, chromosome : 1 (LN847245)	71%	Oligoendopeptidase F homolog (*Chlamydia pneumoniae*) (CRI42262)	72%
adk	*Chlamydophila pneumoniae* LPCoLN, complete genome (CP001713)	67%	adenylate kinase (*Chlamydia pneumoniae*) [ACZ33222.1] (WP_014517853)	62%
hemL	*Chlamydia pecorum* P787, complete genome (CP004035)	67%	glutamate-1-semialdehyde 2,1-aminomutase (*Chlamydia pneumoniae*) [Q9JRW9.1] (WP_010892127)	66%

Supplementary Table S2: Chlamydial contig BLAST analysis

**Contig**	**Contig length (bp)**	**Trimmed length (bp)**	**Top BLASTn hit (accession no.)**	**% ID**
NODE_1	1113133	1113073	*Chlamydia pneumoniae* LPCoLN, complete genome (CP001713.1)	81%
NODE_177	7631	7504	*Chlamydia pneumoniae* LPCoLN, plasmid (CP001714.1)	77%

Supplementary Table S3: Metagenome coverage statistics

Mapping target	No. reads mapped	% of reads mapped	No. contigs	Length (bp)	Mean coverage
Read mapping to *Chlamydia sp.* genome					
Chlamydia sp. 2742-308 chromosome	329886	5.9	1	1113073	44.4x
Chlamydia sp. 2742-308 plasmid	2056848	37.0	1	7504	1888.6x
Read mapping to host microflora genomes[a,i]					
Microflora contigs	176479	3.2	314	11870225	9.4x
Read mapping to host genomic data					
Mitochondrial contigs[b]	271861	4.9	2	17078	1904.4x
Putative chromosomal contigs[c,ii]	n.m	n.m	227252	120657504	n.m

Trimmed reads (5,561,445) were mapped to contigs representing the chlamydial chromosome and plasmid, as well as contigs with BLASTn hits against bacterial genomes representing the microflora of the choana of the snake host, as identified by Metaxa (Bengsston, et al., 2011)

[a] Microflora identified by detection of non-chlamydial 16S rRNA sequences in the contigs using Metaxa. See Table S4 for more details.

[i] Bacterial genomes used: *Elizabethkingia anopheli* s NUHP1 (NZ_CP007547.1), *Caloramator mitchellensis* VF08 (LKHP01000001-LKHP01000053), *Arthrobacter sp* . Rue61a (NZ_CP003203-CP003205).

[b] Mitochondrial contigs identified by detection of 12S rRNA sequences in the contigs using Metaxa. See Table S4 for more details.

[c] Putative genomic contigs identified by BLASTn analysis against an in-house database consisting of two snake genomes.

[ii] Snake genomes used: *Python bivittatus* (AEQU00000000.2) and *Pantherophis guttatus* (JTLQ00000000.1)

n.m; not mapped

Supplementary Table S4: Putative host contig analysis

Putative host contig analysis	
No. contigs BLASTn hit to Snake genomes	227257
No. hits >70% ID and e-value <0.001	227252
Contig length range	128-20848 bp
Mean contig length	530 bp
No. contigs > 1000 bp	21163

Supplementary Table S5: rRNA-containing contigs detected by Metaxa

**Contig**	**Contig length (bp)**	**Top BLASTn hit (accession no.)**	% ID
Bacterial origin			
NODE_1	1113133	Uncultured *Chlamydia sp.* clone 2742-308 genotype 4 16S ribosomal RNA gene, partial sequence (KT012690.1)	99%
NODE_270	5536	*Elizabethkingia anophelis* NUHP1, complete genome (CP007547.1)	97%
NODE_572	3810	Uncultured bacterium clone BJGMM-3s-491 16S ribosomal RNA gene, partial sequence (JQ801102.1)	90%
NODE_626	3673	Uncultured *Segetibacter sp* . clone CNY_00741 16S ribosomal RNA gene, partial sequence (JQ400834.1)	90%
NODE_41381	762	*Arthrobacter sp.* F_172 16S ribosomal RNA gene, partial sequence (KF322151.1)	99%
NODE_185803	315	*Caloramator mitchellensis* strain VF08 16S ribosomal RNA gene, partial sequence (NR_117542.1)	99%
NODE_227855	292	*Arthrobacter sp.* F_172 16S ribosomal RNA gene, partial sequence (KF322151.1)	100%
Mitochondrial origin			
NODE_544	3890	*Eunectes notaeus* mitochondrial 12S rRNA gene,...and tRNA-Val gene (AM236347.1)	84%
