# Supplementary figures and images for: Culture-independent genomic characterisation of Candidatus Chlamydia sanzinia, a novel uncultivated bacterium infecting snakes
Source: BMC Genomics. 2016 Sep 5;17(1):710. doi: 10.1186/s12864-016-3055-x (PMC5011893; doi:10.1186/s12864-016-3055-x)

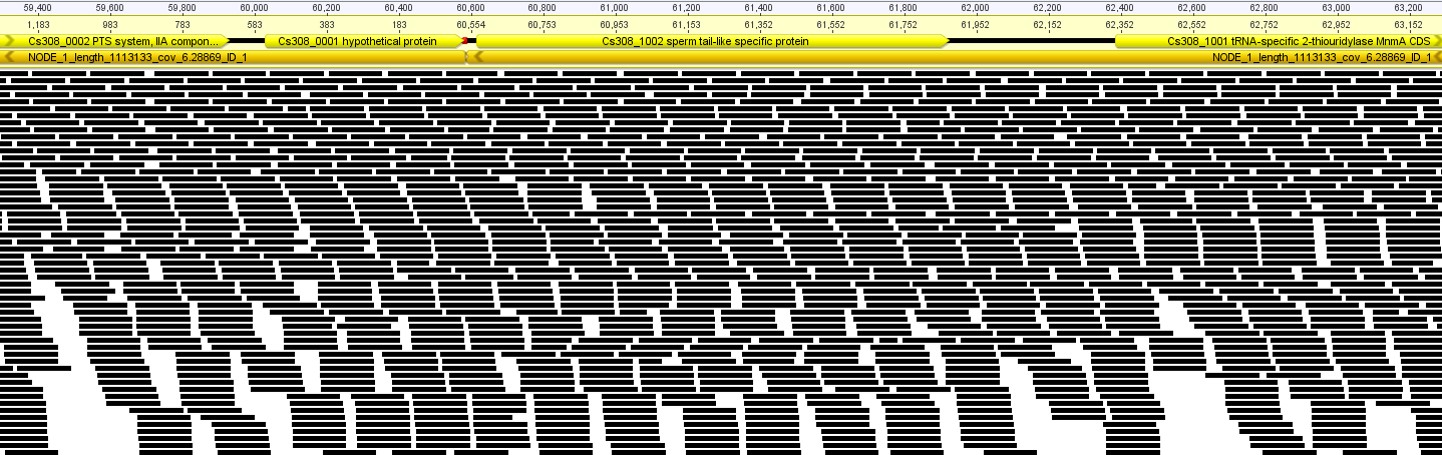

Supplement: Additional file 2: Figure S1. — Contig break mapping. The chromosomal contig was split to resemble the genome architecture of C. trachomatis, and reads were mapped back to the assembly to assess genome coverage in Geneious. A 60 bp region with <10× coverage was removed from the 5’ end of the contig, resulting in reads overlapping the the 5’ and 3’ ends of the contig. (JPG 257 kb) [file 12864_2016_3055_MOESM2_ESM.jpg]

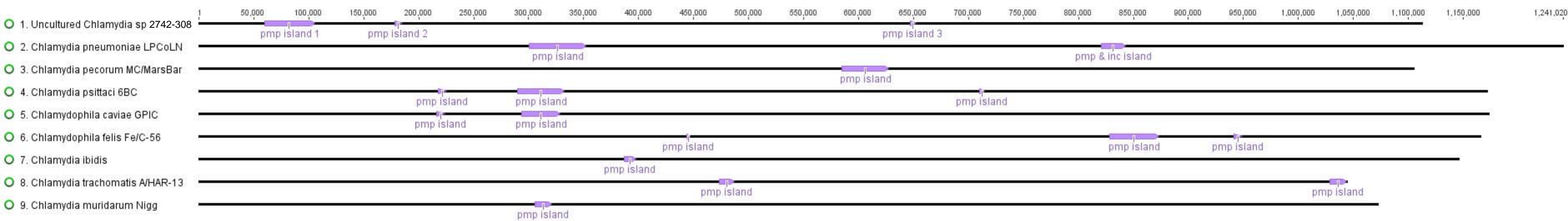

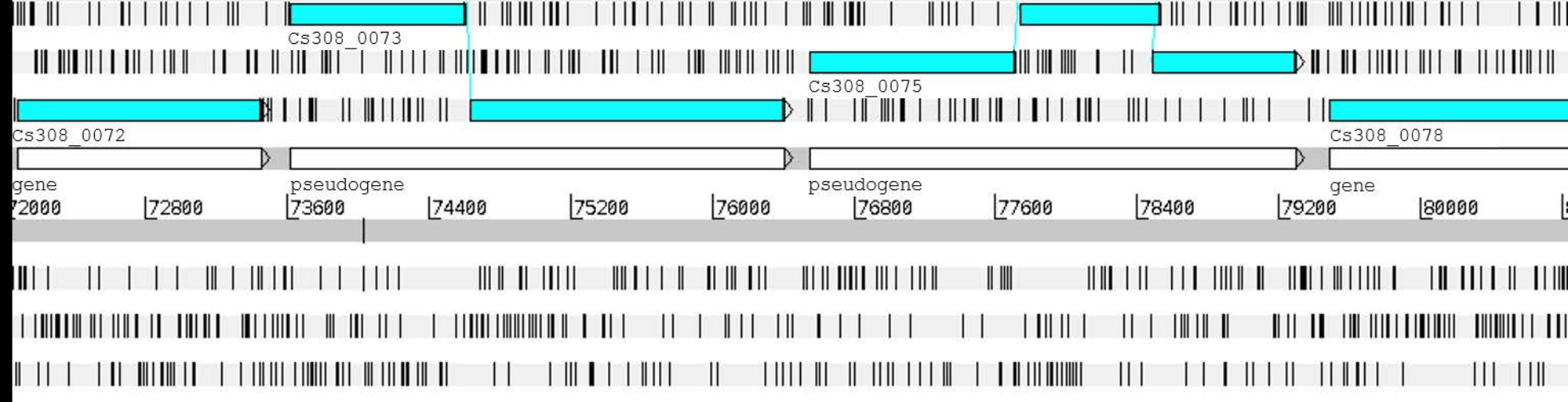

Supplement: Additional file 4: Figure S2. — Locations of “pmp islands” in the uncultured Ca. Chlamydia sanzinia genome and selected chlamydial genomes. Figure was constructed in Geneious. Figure S3. Schematic representation of pmp pseudogenes attributed to frameshift mutations. Open reading frames are coloured blue in frame and genes/pseudogenes are coloured white on the forward strand. Vertical black lines represent stop codons. Diagonal blue lines represent fragmentation due to premature stop codons. Figure constructed using Artemis. (PDF 140 kb) [file 12864_2016_3055_MOESM4_ESM.pdf]
