# Supplementary material for: Culture-independent genomic characterisation of Candidatus Chlamydia sanzinia, a novel uncultivated bacterium infecting snakes
Source: BMC Genomics. 2016 Sep 5;17(1):710. doi: 10.1186/s12864-016-3055-x (PMC5011893; doi:10.1186/s12864-016-3055-x)
Supplement: Additional file 5: Table S14. — Inclusion membrane prediction. (PDF 99 kb) [file 12864_2016_3055_MOESM5_ESM.pdf]

Supplementary Table S14: Inclusion membrane protein prediction

Locus tag	Length (amino acids)	No. amino acids expected in TMH	No. amino acids expected in TMH in first 60 amino acids	Predicted no. of TMHs	Transmembrane topology
G308_0021	len=63	ExpAA=45.74	First60=45.71	PredHel=2	Topology=i12-34o38-60i
G308_0028	len=212	ExpAA=46.65	First60=15.41	PredHel=2	Topology=i47-69o74-96i
G308_0064	len=95	ExpAA=43.70	First60=37.82	PredHel=2	Topology=i5-27o42-64i
G308_0067	len=95	ExpAA=43.86	First60=39.25	PredHel=2	Topology=i5-27o42-64i
G308_0096	len=211	ExpAA=47.73	First60=1.18	PredHel=2	Topology=i102-124o129-151i
G308_0097	len=534	ExpAA=47.66	First60=23.25	PredHel=2	Topology=o32-54i59-81o
G308_0098	len=519	ExpAA=46.73	First60=41.30	PredHel=2	Topology=o15-37i44-66o
G308_0101	len=544	ExpAA=47.83	First60=23.57	PredHel=2	Topology=o33-55i62-84o
G308_0142	len=612	ExpAA=47.17	First60=23.05	PredHel=2	Topology=o35-57i64-86o
G308_0147	len=94	ExpAA=43.89	First60=25.99	PredHel=2	Topology=i31-53o57-79i
G308_0148	len=374	ExpAA=43.81	First60=22.44	PredHel=2	Topology=o36-58i65-87o
G308_0178	len=64	ExpAA=42.49	First60=42.49	PredHel=2	Topology=i7-26o31-53i
G308_0192	len=350	ExpAA=44.49	First60=22.23	PredHel=2	Topology=o36-58i65-87o
G308_0385	len=275	ExpAA=43.95	First60=0.01	PredHel=2	Topology=i222-244o248-270i
G308_0387	len=402	ExpAA=37.41	First60=37.30	PredHel=2	Topology=o15-34i41-58o
G308_0438	len=479	ExpAA=44.52	First60=0.00	PredHel=2	Topology=o252-274i281-303o
G308_0451	len=152	ExpAA=63.23	First60=15.38	PredHel=2	Topology=i48-70o103-134i
G308_0460	len=155	ExpAA=39.64	First60=0.00	PredHel=2	Topology=o98-120i133-150o
G308_0628	len=378	ExpAA=56.73	First60=37.57	PredHel=2	Topology=o15-33i54-76o
G308_0673	len=101	ExpAA=41.33	First60=14.96	PredHel=2	Topology=i45-67o71-93i
G308_0714	len=67	ExpAA=44.93	First60=44.75	PredHel=2	Topology=i7-28o38-60i
G308_0756	len=114	ExpAA=45.22	First60=22.63	PredHel=2	Topology=i35-57o61-83i
G308_0759	len=115	ExpAA=46.57	First60=22.87	PredHel=2	Topology=o38-60i67-89o
G308_0761	len=237	ExpAA=48.55	First60=22.86	PredHel=2	Topology=i37-59o74-96i
G308_0804	len=351	ExpAA=45.63	First60=27.63	PredHel=2	Topology=i30-52o56-78i
G308_0810	len=135	ExpAA=60.98	First60=18.30	PredHel=2	Topology=i80-102o106-128i
G308_0814	len=210	ExpAA=46.05	First60=19.32	PredHel=2	Topology=i40-62o77-99i
G308_0823	len=155	ExpAA=56.24	First60=18.83	PredHel=2	Topology=i50-72o100-122i
G308_0825	len=151	ExpAA=46.49	First60=22.87	PredHel=2	Topology=o31-53i97-119o
G308_0844	len=360	ExpAA=44.46	First60=21.81	PredHel=2	Topology=i38-57o67-89i
G308_0881	len=101	ExpAA=45.42	First60=22.60	PredHel=2	Topology=i36-58o62-84i
G308_0908	len=444	ExpAA=55.91	First60=28.48	PredHel=2	Topology=o27-49i56-78o
G308_0925	len=459	ExpAA=45.79	First60=22.93	PredHel=2	Topology=o34-56i61-83o
G308_0926	len=451	ExpAA=47.18	First60=29.15	PredHel=2	Topology=o26-48i55-77o
G308_0931	len=102	ExpAA=41.06	First60=11.56	PredHel=2	Topology=o49-68i75-94o
G308_0939	len=388	ExpAA=45.87	First60=20.86	PredHel=2	Topology=i38-60o73-95i
G308_0940	len=407	ExpAA=48.71	First60=20.84	PredHel=2	Topology=i40-62o66-88i
G308_0960	len=111	ExpAA=44.27	First60=24.22	PredHel=2	Topology=i31-53o58-80i
G308_0057	len=155	ExpAA=70.47	First60=27.78	PredHel=4	Topology=i13-32o37-59i72-94o99-117i
G308_0764	len=1434	ExpAA=88.85	First60=0.00	PredHel=4	Topology=o73-95i97-116o831-853i858-880o
G308_0909	len=171	ExpAA=88.40	First60=31.42	PredHel=4	Topology=i5-27o47-69i93-115o135-157i

Hypothetical proteins were subject to transmembrane helix prediction using TMHMM (Sonhammer *et al.* , 1998). Proteins with either 2 or 4 TMHs were predicted to be putative Incs.

TMH; Transmembrane helix
