# Supplementary material for: Culture-independent genomic characterisation of Candidatus Chlamydia sanzinia, a novel uncultivated bacterium infecting snakes
Source: BMC Genomics. 2016 Sep 5;17(1):710. doi: 10.1186/s12864-016-3055-x (PMC5011893; doi:10.1186/s12864-016-3055-x)
Supplement: Additional file 6: Table S15. — QUAST report. (PDF 127 kb) [file 12864_2016_3055_MOESM6_ESM.pdf]

Supplementary Table S15: QUAST output

Assembly	trimmed_sc-contigs	trimmed_mc-contigs
SPAdes assembly mode	single cell	multi cell
# contigs (>= 0 bp)	267892	261306
# contigs (>= 1000 bp)	24352	22119
Total length (>= 0 bp)	146043177	139145831
Total length (>= 1000 bp)	42507560	38923074
# contigs	75776	69575
Largest contig	1049755	1113133
Total length	80009657	73660544
GC (%)	37.68	37.59
N50	1045	1040
N75	769	768
L50	21903	20067
L75	44365	40787
# N's per 100 kbp	0	0

QUAST; Quality Assessment Tool for Genome Assemblies
